# Personal

**Published:** 1897-08

**Authors:** 


### Personal.

Dr. B. Sherwood-Dunn, of Los Angeles, (*Pacific Med. Journal*, July,) suffered several weeks ago from a very serious loss by fire. His valuable collection of paintings, bronzes, bric-a-brac, tapestry and an extensive library of 3,500 handsome volumes were completely destroyed. Many of these books were rare and difficult, if not impossible, to be replaced. But we have other than unpleasant things to report of Dr. Sherwood-Dunn. A visit from the Consul-general of France a few days since brought to him the pleasing intelligence that the French Government had decorated him with the Cross of Officer of the Academy. Medical men are not too frequently the recipients of honor such as this, and the profession of the state will sympathise with Dr. Sherwood-Dunn for his losses by fire and at the same time extend sincere congratulations for his recent and well-deserved honors.

---

Dr. Woods Hutchinson, of Buffalo, contributes an article on the Value of pain, to the *Monist*, for July, which is as striking in its homiletic as it is in its scientific point of view. The author draws some profound ethical conclusions as to the function of pain in life and drives his lessons home with great literary and imaginative power.

---

Dr. Francis E. Fronczak, of Buffalo, has established himself at 508 Fillmore avenue for the practice of his profession. He has been appointed visiting physician and surgeon to the Felician Sisters Hospital, Polish Orphan Asylum, and the Old Man's Home at Cheektowaga.

---

Dr. Roswell Park, whose illness we referred to in the June issue of the Journal, is making good progress toward recovery. He is at present at Cushing's Island on the coast of Maine and expects to resume his work in the early autumn.

---

Dr. A. R. Wright, of Buffalo, was elected president of the American Institute of Homeopathy at its recent meeting held in this city. The meeting next year will be held in Omaha, Neb.

---

Dr. C. F. Durand, formerly of Lockport, has recently taken up his residence in Buffalo. His offices are located at 258 Franklin street.

